# Femtosecond Laser Cataract Surgery

**Published:** 2011-04

**Authors:** Sepehr Feizi

**Affiliations:** Ophthalmic Research Center, Shahid Beheshti University of Medical Sciences, Tehran, Iran

Cataract surgery with intraocular lens (IOL) implantation is the most common ophthalmic surgical procedure worldwide. It is also the most common operation for correction of refractive errors, performed over five times more frequently than corneal refractive procedures. In developed countries, phacoemulsification is the predominant form of cataract surgery, accounting for more than 90% of all operations. Although a number of developments have occurred in IOL technology, the basic phacoemulsification procedure has remained largely unchanged over the past 20 years and involves a series of steps including creation of a corneal incision, capsulorrhexis and phacofragmentation.

Femtosecond lasers represent an important technological advance in ophthalmic surgery. Since 2001, several femtosecond laser systems have been introduced and more than 2 million ophthalmic procedures have been performed with these lasers, primarily for the creation of the corneal flap in laser in situ keratomileusis.

With their computer-controlled optical delivery systems, femtosecond laser machines can produce precise surgical incisions without collateral damage to surrounding tissues. Recently the femtosecond laser has received FDA clearance for use in cataract surgery. The femtosecond laser can create a capsulorrhexis measuring a desired diameter, e.g., 5.25 mm, 5.50 mm, 5.75 mm, etc., well-centered on the visual axis without radial tears, resulting in more reproducible positioning of the IOL. Aside from capsulotomy, the laser can perform a three-dimensional configuration corneal cut; this allows multiplanar self-sealing incisions and precise placement of limbal relaxing incisions, potentially increasing the safety and efficacy of cataract surgery. Photofragmentation of the nucleus is also feasible without the risk of damaging the posterior capsule. These lasers will have the ability to break down the lens into fine fragments which are easily removable by aspiration. Importantly, the laser wavelength is not absorbed by the cornea. Unlike the large shock and acoustic waves generated by phacoemulsification devices which are associated with endothelial cell damage, those generated by femtosecond photodisruption dissipate within approximately 100 μm of the target tissue, which is safely located a few millimeters away from the corneal endothelium. However, the use of this technology in eyes with hard nuclei is questionable and requires further evaluation.
